# The Potential Role of Neglected and Underutilised Crop Species as Future Crops under Water Scarce Conditions in Sub-Saharan Africa

**DOI:** 10.3390/ijerph120605685

**Published:** 2015-05-26

**Authors:** Pauline Chivenge, Tafadzwanashe Mabhaudhi, Albert T. Modi, Paramu Mafongoya

**Affiliations:** 1 International Crops Research Institute for the Semi-Arid Tropics, P.O. Box 776, Bulawayo, Zimbabwe; 2 School of Agricultural, Earth and Environmental Sciences, University of KwaZulu-Natal, P. Bag X01, Scottsville 3209, Pietermaritzburg, South Africa; E-Mails: mabhaudhi@ukzn.ac.za (T.M.); modiAT@ukzn.ac.za (A.T.M.); mafongoya@ukzn.ac.za (P.M.)

**Keywords:** biodiversity, sustainability, semi-arid tropics, climate change, food and nutrition security, indigenous knowledge, resilience

## Abstract

Modern agricultural systems that promote cultivation of a very limited number of crop species have relegated indigenous crops to the status of neglected and underutilised crop species (NUCS). The complex interactions of water scarcity associated with climate change and variability in sub-Saharan Africa (SSA), and population pressure require innovative strategies to address food insecurity and undernourishment. Current research efforts have identified NUCS as having potential to reduce food and nutrition insecurity, particularly for resource poor households in SSA. This is because of their adaptability to low input agricultural systems and nutritional composition. However, what is required to promote NUCS is scientific research including agronomy, breeding, post-harvest handling and value addition, and linking farmers to markets. Among the essential knowledge base is reliable information about water utilisation by NUCS with potential for commercialisation. This commentary identifies and characterises NUCS with agronomic potential in SSA, especially in the semi-arid areas taking into consideration *inter alia*: (i) what can grow under water-scarce conditions, (ii) water requirements, and (iii) water productivity. Several representative leafy vegetables, tuber crops, cereal crops and grain legumes were identified as fitting the NUCS category. Agro-biodiversity remains essential for sustainable agriculture.

## 1. Introduction

Biodiversity is fundamental for ecosystem functioning, sustainable agricultural production [1] and the attainment of food and nutritional security [2,3,4], yet only a few crop species are utilised for food production throughout the world [5]. The more diverse farming systems are, the more resilient they are in the face of biotic and abiotic stresses and enhancing food and nutrition security. In addition to provisioning for food, maintaining biodiversity in agriculture is important for providing regulatory ecosystem services such as nutrient cycling, carbon sequestration, soil erosion control, reduction of greenhouse gas emissions and control of hydrological processes. Modern agricultural systems promote the cultivation of high-input and high-yielding crop species, with the intensification of a limited number of species. This has caused a decline in crop diversity in agricultural systems across the world, associated with a diminishing of the regulatory services. Of particular concern, the cultivation of traditional crops has declined and continues to decline globally, yet such crops offer greater genetic biodiversity, and have potential to improve food and nutritional security. This is particularly important to ensure food and nutritional security for the current increasing population in a world of finite resources.

“Food and nutrition security exists when all people, at all times, have physical and economic access to sufficient, safe and nutritious food that meets their dietary needs and food preferences for an active and healthy life” [6]. The Millennium Development Goals (MDG-1) that expire at the end of 2015 spelt out the importance of food and nutrition security. Although progress has been made towards achieving MDG-1 by 2015, sub-Saharan Africa (SSA) retains the unenviable position of being the region with the highest prevalence of undernourishment [7]. The prevalence of undernourishment in SSA currently stands at 23.8% with most countries being characterised as food and nutrition insecure [8]. In a region where 70% of the population relies on agriculture, it follows that agriculture remains the main vehicle for addressing food and nutrition security. The approach taken during the last decades was to promote the cultivation of a few high yielding high input crops. While this has helped to reduce levels of food insecurity, it paid lip service to nutritional security due to focus on a few starchy crops. The need for nutritional security cannot be understated; it is the foundation upon which human well–being is built [9]. Hence, despite gains having been made over the last 15 years, MDG 1 has not been fully met. It is this realisation that has also informed the soon to be ushered in Sustainable Development Goals (SDGs) which will replace the MDGs post 2015 (2016 to 2030).

As the world ushers in the SGDs, there is need to rethink strategies. An alternative strategy is tapping into SSA’s agro-biodiversity and broadening the food basket to meet the nutritional requirements of people in the region. Traditional crop species which are often neglected and underutilised, rely on biological functioning of the ecosystem, require low input of synthetic fertilisers, pesticides and irrigation could be promoted as an alternative for ensuring food and nutritional security [10,11]. Diversity of diets based on diverse crops delivers better nutrition and greater health with additional benefits for human productivity and livelihoods.

## 2. Climate Change, Water Scarcity and the Concern to Sustainability of Food Production and Food Security

The greater parts of SSA are classified as semi-arid and are found in the drylands characterised by frequent drought occurrences in many seasons. This, coupled with climate change and variability has resulted in enormous negative effects on local food production and food and nutritional security. This is more important in SSA where a greater part of the population depends on smallholder agriculture, particularly for groups with low income and low adaptive capacity facing significant threats to food security.

According to the Intergovernmental Panel on Climate Change (IPCC), SSA is one of the global regions that are most vulnerable to climate change and variability [12,13]. This is aggravated by the fact that SSA is also one of the global regions with the least adaptive capacity to climate change [13,14]. Current climate change predictions for the region paint a scenario of rising temperatures, increased variability in rainfall (change in patterns, onset and amounts) and increased frequency of extreme weather events such as drought and floods [12,13]. Already, SSA is experiencing some of these weather extremes with drought and floods ravaging most of SSA’s landscape. While the threat of climate change and variability straddles most sectors of SSA’s economies, agricultural production is of particular concern. Most of SSA’s economies are still heavily dependent (directly or indirectly) on agriculture as a driver of economic and rural development. Most importantly, agriculture remains a source of livelihood and food security for the majority of the SSA’s population with about 95% of agriculture being rainfed [15] and subsistence based. This is of great concern when viewed within the context of the impacts all this will have on agriculture, and the vulnerability of rural households and the urban poor, regarding food and nutrition security, because the incidence of crop failure will likely increase [16].

According to Schulze [17], water is the primary medium through which impacts of climate change and variability will be experienced. This will place further strain on the SSA’s already limited water resources. Under these conditions, food, nutritional and income insecurity, which are already a challenge across much of SSA, [7,18], may be exacerbated. Smallholder farmers, who lack the resources, to adapt and respond to the effects of climate change will particularly feel these pressures. Given the continued importance and potential of agriculture within SSA, there is an urgent need to develop strategies that can ensure the viability of this key group of farmers.

Consequently, improving agricultural productivity still remains an important feature of SSA’s development agenda. Current strategies have mainly centred on crop improvement of a limited set of major crops. While these strategies have played a major part in addressing food security, they have been unable to resolve SSA’s nutritional challenges. This has led to the region failing to achieve Millennium Development Goal Number one [9]. Already there are suggestions that some of these crops may not be able to adequately ensure food and nutrition security, particularly under the predicted climate change [19,20] This is especially true for much of SSA where climate change and variability threaten gains made in food security. As we usher in the Sustainable Development Goals (SDGs), there is also a need to reconsider approaches to ensuring food and nutrition security [21]; it certainly cannot be business as usual.

There is a need for “new” and/or “alternative” approaches to ensuring food and nutrition security. Such solutions should be sustainable, resilient and of practical solutions to challenges facing SSA’s smallholder farmers, particularly increasing water scarcity due to climate change and variability. This need has led to renewed focus on identifying and improving underutilised indigenous and traditional crops for drought tolerance [22]. Biodiversity is essential to cope with predicted impacts of climate change and increase pests and diseases under climate change and variability.

## 3. Neglected and Underutilised Crop Species (NUCS)

Given the above background of limited water resources, the perceived threat of climate change and the need to come up with mitigation strategies, this commentary aims to highlight some of the progress made in developing and increasing the pool of available information describing drought tolerance and water-use of selected neglected and underutilised crop species. In the subsequent sections, this commentary will seek to describe what underutilised crops are, the diversity they represent, their current status in terms of utilisation as well as their known drought tolerance.

Currently there is a lack of a consensus definition for neglected and underutilised crop species. There is even a lack of consensus on what these crops should be referred to as with different names referred to by different names e.g., orphan crops, neglected crops, underutilised crops, forgotten crops, minor crops, *etc.* For the purpose of this review we refer to this collective group as neglected and underutilised crop species (NUCS) and define them as crops that have not been previously classified as major crops, have previously been under-researched, currently occupy low levels of utilisation and are mainly confined to smallholder farming areas [23]. Historically, such crops have played an important role in ensuring community and household food and nutrition security through providing healthy alternatives when the main crop failed or during periods in-between subsequent harvests [19]. Promotion of NUCS, with a view to reinstating them as alternative food sources in agriculture will depend, to a large extent, on availability of information describing their agronomy, water-use and possible drought tolerance.

There is currently limited literature describing growth, development and water-use aspects of traditional and indigenous crops. Such information, when it exists, is often locked up in indigenous knowledge systems and other grey literature which are not easily accessible. In addition, there has not been much coordination on studies related to NUCS, both on a regional scale as well as internationally. Globally, there have been such studies, for example the BAMLINK project and recently the Edible Aroids project, which have attempted to improve knowledge on agronomy and genetics of bambara groundnuts, and root and tuber crops, respectively. The lack of coordination and non-uniformity of an umbrella term could also partly explain why there is little information available in search engines on NUCS.

According to Garn and Leonard [24], between 300,000 to 500,000 plant species exist, out of which 30,000 are thought to be edible. Throughout history, of the 30,000 edible plants, only 7000 have been either cultivated or collected as food. Of even greater concern is the fact that only 20 species have provided for 90% of the world’s food requirements [25], with wheat, maize and rice accounting for 60% of man’s diet [25]. Thus, tens of thousands of edible plant species remain relatively “underutilised”, with respect to their ability to contribute to the world’s increasing food requirements. Consequently, there has been a reduction in genetic diversity underpinning agriculture; this is accompanied by the displacement of indigenous species by more favoured major crops [23]. The displacement of NUCS can be attributed to several factors which include under-research, lack of information on their production and socio-economic factors that influence food choices among others. However, as Prescott-Allen and Prescott-Allen [26] highlighted, the importance of many indigenous species should not be neglected; this is because of the genetic diversity that underpins them as well their adaptation to ecological niches [27].

Unlike most staple crops, NUCS are often well–adapted to local growing conditions [27], which are often marginal and harsh, thus offering sustainable food production [28]. Neglected underutilised crop species that are common among SSA’s farming systems include many *Amaranthus species* [29], wild mustard (*Brassica* spp.) and other wild edible leafy vegetables [30] as well as sweet potatoes (*Ipomoea batata*), wild melon (*Curcubita* spp.), taro (*Colocasia esculenta*) and bambara groundnut (*Vigna subterranea*), to mention just a few. Historically, these crops have provided dietary support to indigenous communities. However, cultivation of NUCS has become non-competitive and unattractive compared to the “major” crops, which are promoted even in less suitable areas at times. The promotion of “major crops” has been achieved through the formal seed systems and markets that serve them as well as availability of extension support for farmers.

Across much of SSA, water availability remains the major limiting factor to crop production, with limited infrastructure and technical knowledge for irrigation development, threatening food security of vulnerable groups. Additionally, a greater proportion of land in smallholder farming systems is degraded. Most NUCS are believed to be adapted to a range of ecological niches, low input agriculture and may have tolerance to abiotic and biotic stresses. Neglected underutilised crop species are often described as “drought tolerant” [31] and could therefore prove vital in fighting hunger. This makes them important future crops for SSA’s smallholder farmers on marginalised lands especially under water-scarce conditions. As such, the importance of NUCS should not be underestimated [26].

However, limited information describing basic aspects of their genetic potential, agronomy, water requirements and nutrition remains a hindrance to their development and promotion. Such information may be available in “grey literature” and/or indigenous knowledge systems, both of which are unavailable to a greater audience. Of particular interest is indigenous knowledge which is primarily responsible for in-situ conservation of most of NUCS. This review focusses on examples of a few selected NUCS where progress has been made in this regard.

## 4. Tapping into Indigenous Knowledge

An important source of resilience for indigenous people is their ability to nurture and manage domestic and agrobiodiversity, recognizing that crop success is subject to variability and unpredictability of weather events and occurrence of pests. Rao [32] argued that the basis of any society’s knowledge system was built on indigenous knowledge (IK). It may thus be argued that the basis of NUCS knowledge systems is also closely tied to IK. This is because NUCS have been grown, utilised and conserved within smallholder farming communities in SSA. Indigenous communities have traditionally favored cultivation of diverse traditional crop varieties/landraces (NUCS) and over monocropping, which is risky. As such, much knowledge about the utilisation and intrinsic value associated with NUCS remains hidden in the IK of these communities. The marginalisation of NUCS due to the introduction of a limited number of industrialised crops has led to the loss of IK of such crop species.

Indigenous knowledge is an important part of people’s capacity to conserve and manage natural and agricultural ecosystems [33]. This knowledge is acquired through frequent interactions with the local environment driven by the need to pursue subsistence strategies for food and economic provision. This knowledge is transferred from generation to generation through observations and narrations as a key survival tool. It is socially embedded, contributing to cultural traditions, identities, beliefs and world views. It differs from modern knowledge by being dynamic, locally derived and thus co-evolving with the ecosystem upon which it is based [34]. The importance of IK should be emphasised in the design and implementation of development projects, and should be incorporated in research on NUCS. Incorporating IK in research and development of NUCS would help to steer away from top-down development strategies [35].

Traditional farmers have domesticated, improved and conserved thousands of crop species and varieties [36]. There is abundant evidence that communities and farmers using IK are already involved in selecting new varieties/landraces and adopting new crops. In Niger and Mali the amounts of intra-crop diversity of traditional varieties of pearl millet and sorghum have remained broadly similar throughout the dry periods for the last 30 years. This suggests that these materials show sufficient adaptability to enable farmers to cope with periods of significant rainfall shortage [37]. In both countries there was a loss of long duration types of pearl millets and sorghum with a preference for early maturing varieties. The increasing importance of traditional crops is shown in other parts of the world, e.g., Northern India where there is dependence on finger millet. For the past four years rainfall has been decreasing to 300 mm, yet the finger millet varieties grown and conserved by the farmers have excellent drought resistance trait [38]. Hence resilience is rooted in traditional knowledge of indigenous people.

Studies by Swiderska, Reid [36] assessed the role of IK and related agro biodiversity for adaption to climate change results showed traditional maize varieties used in South west China were drought and wind resistant. Similarly, traditional maize varieties used in Kenya were resistant to unpredictable weather events and pests, and potato varieties used in Bolivia were more resistant to new pest and low rainfall. Shava, O’Donoghue [39] discussed the management of diversity in its multiple aspects among farming communities in Zimbabwe. Farmers fostered diversity in order to guarantee a harvest and also to fulfill social and cultural means. These included early maturity maize varieties with local names such as *mukadzi usaende* or *mukadzi dzoka* (these words literally say to a wife: don’t go or wife come back). These varieties were suitable for short rain seasons with intermittent dry spells. The conservation of these landraces also applied to sorghum, pearl millet, cowpea and Bambara groundnut. The growing of different varieties/landraces of the same crop is said to better guarantee a harvest regardless of seasonal variability (short season and long wet season) and to ensure dietary diversity with better nutrition [39,40].

These studies show close interlinkages between IK and genetic resources conservation and their role in adaptation to climate change and variability. This suggest the need to support initiatives such as local landrace conservation, local landrace production, seed fairs, community seed banks and community based conservation and adaptation. The studies also show the role of traditional knowledge and traditional crop varieties in adaptation to climate change.

Thus, there is a need to tap into IK, which may better inform the scientific understanding of the role of NUCS, in relevant ways for local farming systems. Opportunities exist for the poor, especially female headed households, to improve their food security and nutrition through improved utilisation of NUCS if there are concerted efforts to improve the agronomy of such crops in home gardens and fields. The value of NUCS has potential to improve on poverty alleviation, and contributing to health and medical benefits of the local communities [41].

## 5. Drought Tolerance in Selected NUCS

While there have been a whole host of studies on drought tolerance of major crops, there have been much fewer studies describing drought tolerance and water use of NUCS. Where such efforts have occurred, they have been at a much smaller scale, mostly in efforts to study major crops. If NUCS are expected to make a significant contribution as future crops under water limited conditions, this information will need to be generated and made available, at a faster rate than was done for major crops. This commentary focusses on selected NUCS that have been characterised for drought tolerance. As a way of introducing this topic, we have decided to first describe some concepts related to drought tolerance. A plant's chosen mechanism to coping with stress is based on the choice of responses it adopts in responding to developing water stress. Based on this combination, and the magnitude and timing of stress [42], a plant may escape, avoid, and/or tolerate stress.

Drought escape is associated with timing of key phenological stages. Plants that escape drought achieve this by having a short growing season, hence allowing them to complete their growth cycle before water stress becomes terminal. According to Araus, Slafer [43], flowering time is an important adaptation related to drought escape.

The essence of drought avoidance is to reduce water loss while enhancing or maintaining uptake by the roots. Drought avoidance involves crop responses such as stomatal regulation, enhanced capture of soil water through an extensive and prolific root system [44,45]. Several root characteristics such as biomass, length, depth and thickness (volume) are thought to contribute to final yield under drought stress [44,45,46] due to improved water capture. Additionally, reduced water loss by the plant can be achieved by morphological changes: reduced plant height, leaf number, leaf area and leaf area index contribute to reducing water loss by the plant [47] thereby assisting the plant to avoid drought. Blum [42] also associated drought avoidance with reduced season duration due to reduced leaf number; reduced season duration is also characteristic of drought escape, suggesting that the mechanisms do not work in isolation.

Lastly, there is drought tolerance which has been defined as the plant’s capacity to maintain metabolism under drought stress [42]. It includes osmotic adjustment (accumulation of metabolites, osmoprotection (e.g., proline) and the antioxidant defence systems [48]. Blum [42] gave a detailed account of increasing evidence suggesting a relationship between high osmotic adjustment and maintenance of biomass and yield under stress. Unlike escape and avoidance, the *modus operandi* of drought tolerance does not show any solid evidence of a yield reduction [42]. However, drought tolerance as an effective crop drought-resistance mechanism is rare; it mainly exists in seed embryo and is lost after germination [42].

Below we discuss drought tolerance and water use of selected NUCS. These have been purposely selected to represent the broad categories of cereals, legumes, root and tuber crops and leafy vegetables. The basis of this organisation was to also highlight the diversity of NUCS and their ability to contribute to human health and nutrition. In addition to being drought tolerant, the range of NUCS discussed below have the potential to address energy (protein) and mineral deficiency in the diets of people in SSA practising rainfed agriculture. The Global Nutrition Report [9] indicated that these were mainly lacking in the diets of people in the region.

### 5.1. Cereal Crops

#### 5.1.1. Maize Landraces

Of the many crops grown in SSA, maize (*Zea mays* L.) is one of the staple foods. Maize belongs to the family Poaceae (Gramineae) and the tribe Maydeae [49]. Although maize may have its ancestry outside of Africa, it has been around for so long and has become “indigenised” as a result of hundreds of years of farmer and natural selection. Early Portuguese merchants introduced maize into Africa through their trade networks along the eastern and western coasts of Africa starting in the 16th century. The Dutch introduced maize along the southern African coast in 1658 [50].

**Figure 1:**
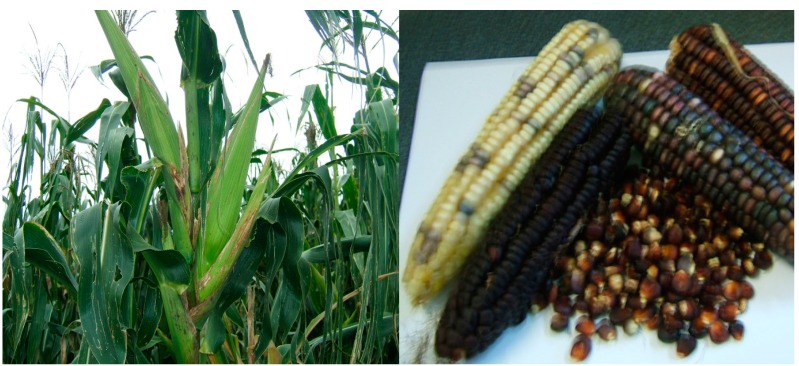
Maize landraces still show much variation with regard to seed colour and ear prolificacy. Source [10].

These varieties formed the now local maize populations or landraces (Figure 1). Zeven [31] defined landraces as crop genetic resources that have evolved continuously under natural and farmer selection practices rather than in the collection of gene banks or plant breeding programs. Historically, landraces were the progenitors of modern crop varieties. Smallholder farmers in traditional farming systems across SSA continue to cultivate maize landraces which they have kept from generation to generation. Although these farmers are still planting maize landraces to this day, there has been little or no research to characterise these landraces with respect to drought tolerance and adaptability to water stress. In a report by Modi and Mabhaudhi [10], it was stated that maize landraces were drought tolerant during the establishment stage and suited to low input agricultural systems. They concluded that the fact that smallholder farming communities continued to cultivate maize landraces despite the low yields, suggested that they possessed other characteristics that made them desirable. Much of these desirable characteristics exist in the IK of the communities that still cultivate them. This reaffirms the need to incorporate IK into efforts to promote NUCS.

#### 5.1.2. Millets

**Figure 2:**
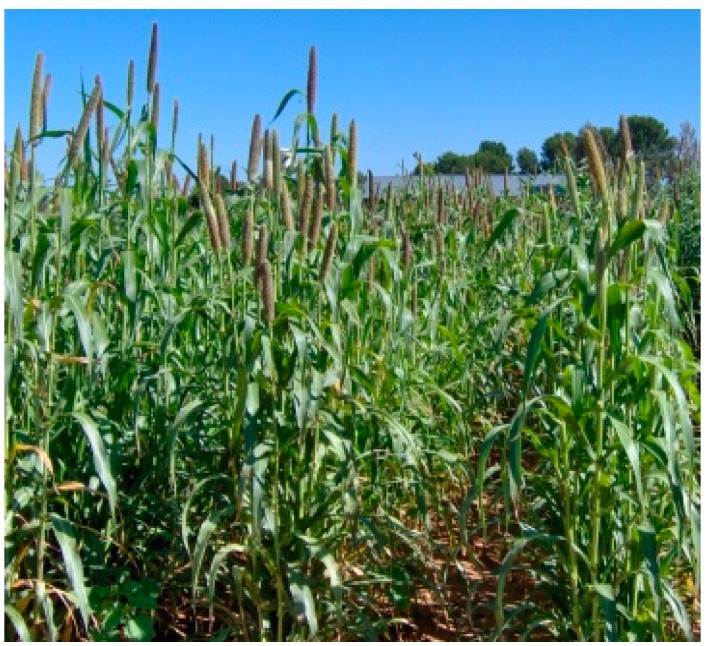
A crop of pearl millet. Source [10].

Millets (pearl, foxtail and finger millet) are an example of indigenous cereals grown in the dry parts of SSA. These crops may have been indigenised to the dry areas due to many years of cultivation, as well as natural and farmer selection. However, now the production of millets is limited to certain areas that are not considered as cereals producing areas in SSA [51]. Across much of SSA, cultivation of pearl millet (Figure 2) is mainly practised at a subsistence level by smallholder farmers. It is only grown commercially as forage for animal consumptions in some areas [52]. Millets are an annual C4 plant that can grow on a wide variety of soils ranging from clay loams to deep sands but the best soil for cultivation is deep, well-drained soil. This makes it suitable for cultivation by smallholder farmers in semi-arid areas where deep sands and sandy loam soils dominate. In addition, millets are easy to cultivate and can be grown in arid and semi-arid regions where water is a limiting factor for crop growth [53,54].

Millets are often referred to as a “high-energy” cereal as they contain higher oil content than maize grains; their protein and vitamin A content are also higher than maize [56,57]. The fact that millets contain vitamin A, a major deficiency in staple diets, makes it a suitable crop for combating nutritional challenges in these communities. Compared with other staple grains such as maize, wheat and sorghum, pearl millet is less susceptible to pests and diseases [56]. Studies on drought tolerance strategies of pearl millet include that of de Rouw [58] and de Rouw and Winkel [59]. They found that the best strategy to reduce risk was spreading of sensitive stages of the crop’s development in order to avoid the hazards of drought that occur during the season. In the case of early relief of drought, recovery of leaf growth supports good grain filling in productive tillers in order to limit the yield losses in the main shoot of pearl millet [60]. This makes millets suited for production under climate change and variability where variability in rainfall will probably expose crops to intermittent stresses. In such cases, millets could be promoted as part of climate change adaptation strategies in areas experiencing huge rainfall variability.

### 5.2. Root and Tuber Crops

#### 5.2.1. Sweet Potato

**Figure 3:**
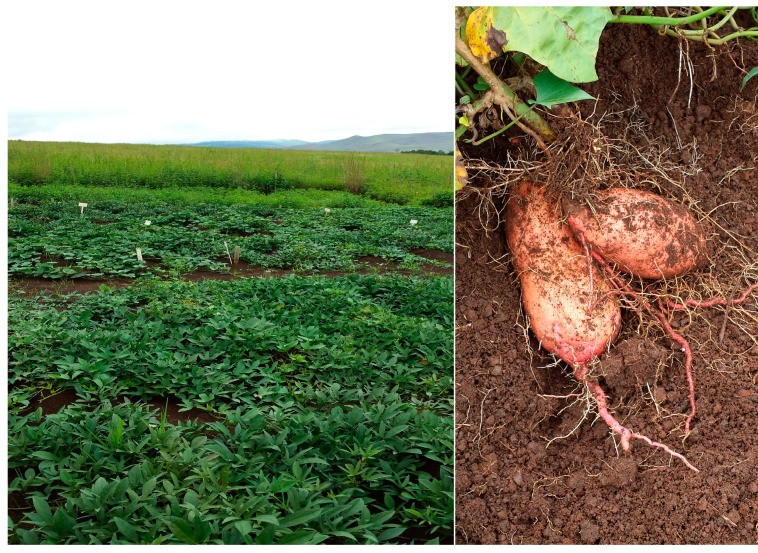
Sweet potato.

Although sweet potato (*Ipomoea batatas*) (Figure 3) is among the earliest first staple crops domesticated by man prior to the introduction of cereal, it still remains one of the NUCS. Together with cassava, sweet potato, yams and aroids are important crops within developing countries [61]. Early Portuguese explorers are believed to have first introduced sweet potato to Africa in the 16th century [61]. Since then, it has spread throughout the continent. It is commonly referred to as the ‘poor man’s crop’; this negative perception may, in part, explain its current status as a NUCS.

Despite this status, sweet potato remains an important root crop of the tropics owing to its versatility [62]. This is with regards to its suitability to low input systems, drought tolerance and large environmental plasticity which allow it to be planted and harvested at any time of the year, especially in frost free areas [63]. Within the communities that consume it, both the leaves and root are utilised for human and animal consumption with limited industrial use [62,64]. Its versatility make it an ideal food security crop [65] capable of contributing to the food and nutritional security of smallholder farmers residing on marginal production lands [66].

Perhaps the biggest contribution of sweet potatoes lies in the potential of the orange-fleshed sweet potato varieties, which are reported to contain significant concentrations of β-carotene, a precursor for vitamin A. As such, orange-fleshed sweet potato varieties are seen to offer potential to contribute significantly towards Vitamin A deficiency; the nutritional dimension of food security.

Studies conducted by Low *et al*. [65] in sub-Saharan Africa established that incorporation of orange-fleshed sweet potato varieties in diets of children led to an improved vitamin A status. Amagloh *et al.* [67] concurred that due to their relatively high levels of vitamin A, orange-fleshed sweet potato varieties could be used as a complementary food for feeding infants. Several studies by Kulembeka *et al.* [68], Laurie and Magoro [69] as well as Laurie and van Heerden [70] reported good acceptability of orange-fleshed sweet potato varieties including the leaves. However, more work still needs to be done to improve on acceptance and utilization.

#### 5.2.2. Taro

**Figure 4:**
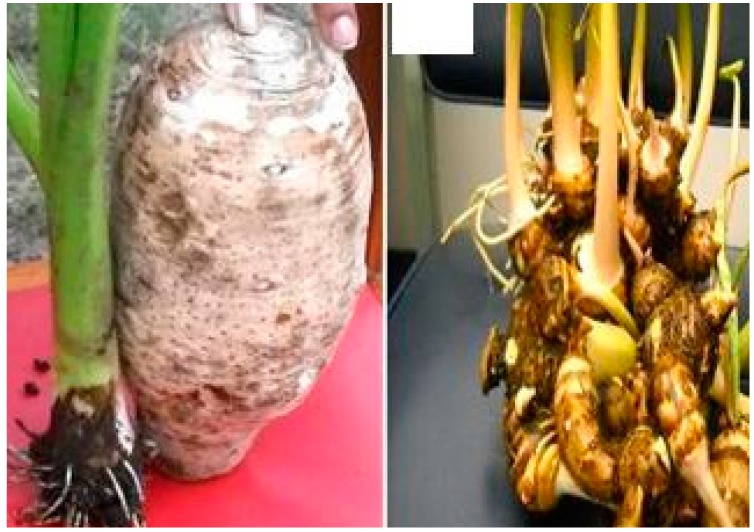
Taro landraces: (**Left**) Var. esculenta—dasheen with one main corm and a huli used as planting material and, (**Right**) Var. antiquorum—eddoe with numerous side cormels. Source [10].

Taro [*Colocasia esculenta* (L.) Schott] (Figure 4) belongs to the family *Araceae*, sub-family *Aroideae* [71]. It is one of the few edible species in the genus *Colocasia* [72] and is the most widely cultivated species [73]. Leaves and corms of taro are edible and are a rich source of carbohydrate, vitamins A and C, and protein. Thus, taro can also serve as a leafy vegetable supplying mineral nutrients to diets of smallholder farmers. Taro also features in several agro–forestry systems due to the fact that it is shade tolerant [74]; this makes it ideal for SSA’s mixed cropping systems which typically feature trees as well. In South Africa, for example, there has been an increase in taro production owing to improved access to niche markets [75,76,77]. Much of this success involved combining IK with science to improve taro production and linking farmers with markets [75,76]. A few studies [55] have now explored the possibility that taro could compliment Irish potato (*Solanum tuberosum*) as an alternative for making crisp chips. This success story is testament to what can be achieved for NUCS when IK is incorporated into developmental strategies targeting them.

As with other NUCS, there have been limited local studies investigating the drought tolerance and water-use of some of the landraces currently being cultivated. With improved information availability, taro production as well as its commercialisation may be expanded beyond current levels. Recently there have been studies indicating that some upland South African taro landraces were drought tolerant [74] and adapted to low levels of water use [78]. While more certainly needs to be done, these results open the door to taro being cultivated beyond the traditional wet areas where it has been produced. Taro is already known to be tolerant to waterlogging [74,78]; with reported moderate drought tolerance it could become an ideal crop for dry areas that are predicted to experience incidences of flash floods.

### 5.3. Grain Legumes

#### 5.3.1. Bambara Groundnut

**Figure 5:**
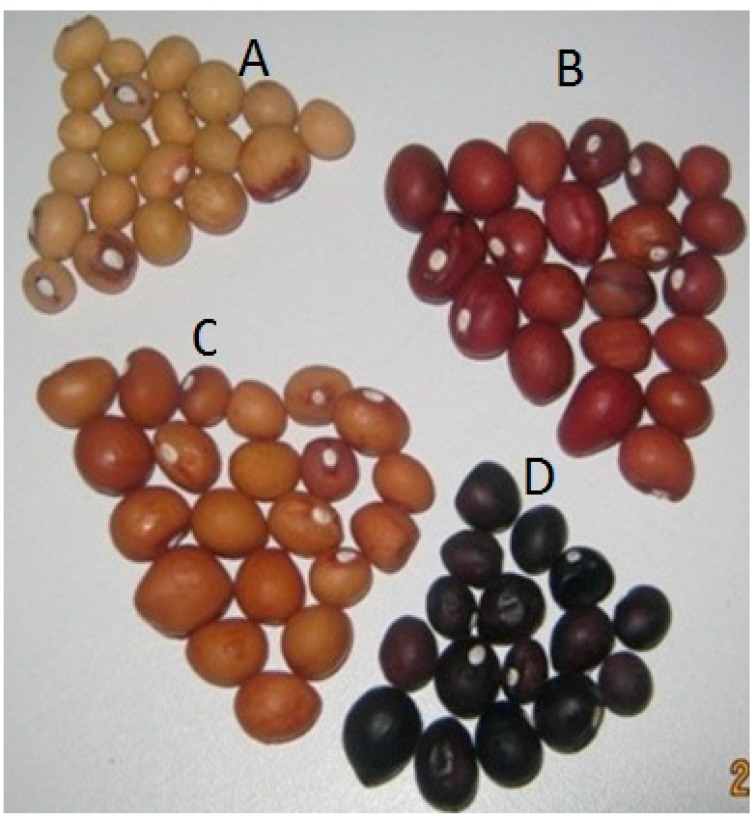
A single Bambara groundnut landraces characterised on the basis of seed coat colour; **A**—Light-brown, **B**—Red, **C**—Brown, and **D**—Black. Source [10].

Bambara groundnut (*Vigna subterranea*) (Figure 5) originated in North Africa and migrated with indigenous people to southern Africa. It is an annual legume with a strong well-developed tap root system. The name originates from Bambara, a district on the upper Niger near Timbuctoo. Traditionally, bambara groundnut was cultivated, mainly by women [79], in semi- and arid regions [80] where water is usually in short supply, without access to irrigation and/or inorganic fertilizers and with little guidance on improved agronomic practices. It has been produced mainly for the sustenance of families locally. Within these communities, bambara groundnut played an important role as a protein source [81]. Its protein content (16%–25%) is comparable, and in some instances, superior to other established legumes, making it a good complement for cereal-based diets [80,81]. As a legume, bambara groundnut also replenishes nitrogen in the soil through nitrogen fixation, an ability that may be of importance to resource-constrained farmers who may otherwise not be able to afford inorganic nitrogen fertilizers. Thus, it is an important crop to incorporate in rotations with cereal crops.

However, due to the expansion of groundnut (*Arachis hypogea*) production, bambara groundnut has been relegated to the status of an underutilised crop in most parts of SSA [82]. As such, its germplasm improvement and agronomic management practices have mainly relied on local experience and resources, *i.e.*, IK [79]. Bambara groundnut is widely reported to be drought tolerant [78,80,83,84] and is, perhaps, one of the NUCS that have received some significant attention with regards to their drought tolerance.

However, that said, the amount of research on bambara groundnut still lags behind that of its erstwhile counterparts such as groundnut and dry bean (*Phaseolus vulgaris*). Its reported drought tolerance and low levels of water use have potential to make it an ideal crop for cultivation in semi-arid areas of SSA that face an increased frequency and intensity of droughts due to climate change.

#### 5.3.2. Cowpea

**Figure 6:**
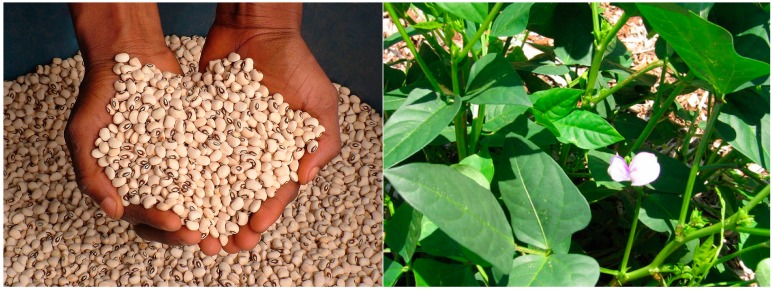
Cowpea seed (**Left**: black-eyed cowpea) and a cowpea plant (**Right**) at the flowering stage of growth. Source [10].

Cowpea (*Vigna unguiculata*) (Figure 6) is a legume crop that belongs to the *Fabacea* family formerly known as *Leguminosae* [85]. It is one of the oldest crops known to man with its centre of origin and domestication being closely related to pearl millet and sorghum in Africa. Cowpea is an important legume which serves as an important source of protein in the diets of vulnerable populations [86]. It is a warm season, annual, herbaceous crop of either an erect, semi-erect (trailing) or climbing growth habit. Cowpea thrives in arid and semi-arid conditions and is produced in areas with optimum rainfall conditions of 400 to 700 mm per annum [52]. Leaves can be consumed as vegetables, while seeds are eaten in the same manner as dry beans. In this instance, cowpea when utilised both as a leafy vegetable and grain legume, can address plug the hunger gap that often plagues farmers during periods before the next harvest. When used in this way, it has significant potential to contribute towards food and nutrition security by providing vitamins and minerals (leaves) [87], and protein (grain), [88]. Cowpea is also commonly used for pastures and fodder, especially in South Africa [10].

Cowpea has a long taproot, reaching a maximum effective rooting depth of about 2.4 m within eight weeks after planting, which proves beneficial in the event of drought and nutrient mining. Research on cowpea has recently started to emerge; however, it is still considered as a NUCS based on social and economic restrictions imposed on its production. Although it been widely reported to be drought tolerant [10], there is limited research being done on the crop. The dual purpose nature of the crop make it an important crop for inclusion in food and nutrition security as well as climate change adaptation strategies for SSA.

### 5.4. African Leafy Vegetables

#### 5.4.1. Amaranth

**Figure 7:**
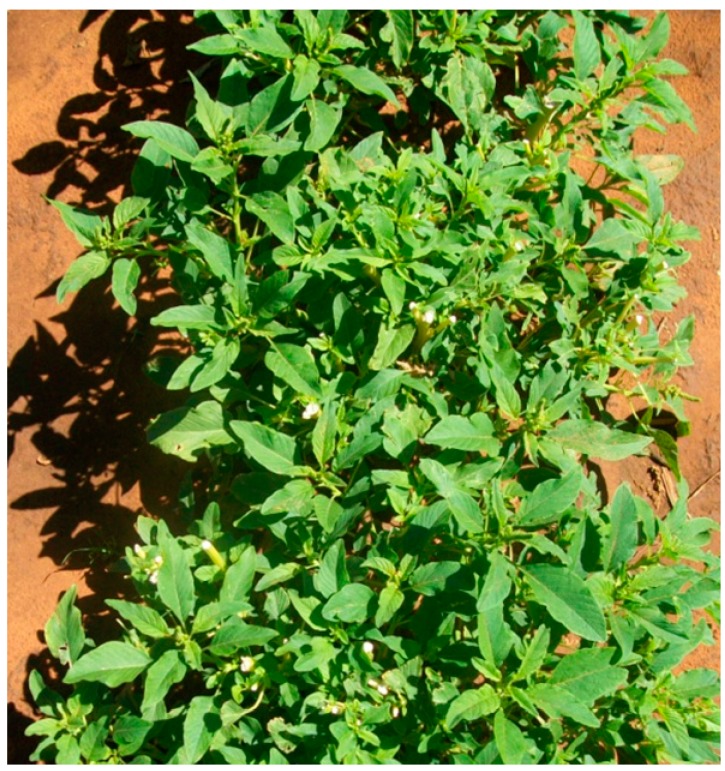
*Amaranthus cruentus*. Source [10].

Amaranth (*Amaranthus* spp) (Figure 7) is an annual C4 crop that grows optimally under warm conditions [89,90]. In southern Africa, amaranth is rarely cultivated because of the belief that it grows naturally, although it has potential to be developed as a cultivated crop [11]. The leaves of amaranth have high protein, vitamins and mineral content [91]. The protein content in the weedy species of amaranth is comparable to the World Health Organisation standards [92]. In addition, amaranth is also a rich source of dietary fibre and lipids rich in unsaturated fatty acids as well several minerals, vitamins and bioactive compounds [93]. Amaranth is considered as a promising crop for cultivation in marginal, arid and semi-arid regions because of its nutritional benefits and ability to adapt to adverse environments [94]. It can grow on a wide range of soils and can tolerate soil pH from 4.5 to 8.0 [95].

*Amaranthus* species are known to be tolerant to adverse climatic conditions [96,97]. A recent review by Alemayehu *et al.* [93] reported that owing to its drought tolerance, promotion of amaranth cultivation as an alternative crop could be vital to combating food and nutrition security under climate change. Amaranth is also known to be moderately tolerant to salinity stress which can help the plant in semi-arid regions as well as areas prone to salinity stress [98]. One of the strategies used by the crop to tolerate salinity is efficient use of water. Rapid leaf area development and high stomatal conductance, rapid root and shoot growth after emergence are part of the features that ensure the crop uses available soil water efficiently [99]. Though, amaranth can cope with adverse conditions, supplementary irrigation and fertilization will increase fresh and dry biomass [100]. The fact that in SSA cultivation of amaranth is limited in extent and scale means that there is also limited information describing drought tolerance and water-use of local *Amaranthus* species.

#### 5.4.2. Wild Mustard

**Figure 8:**
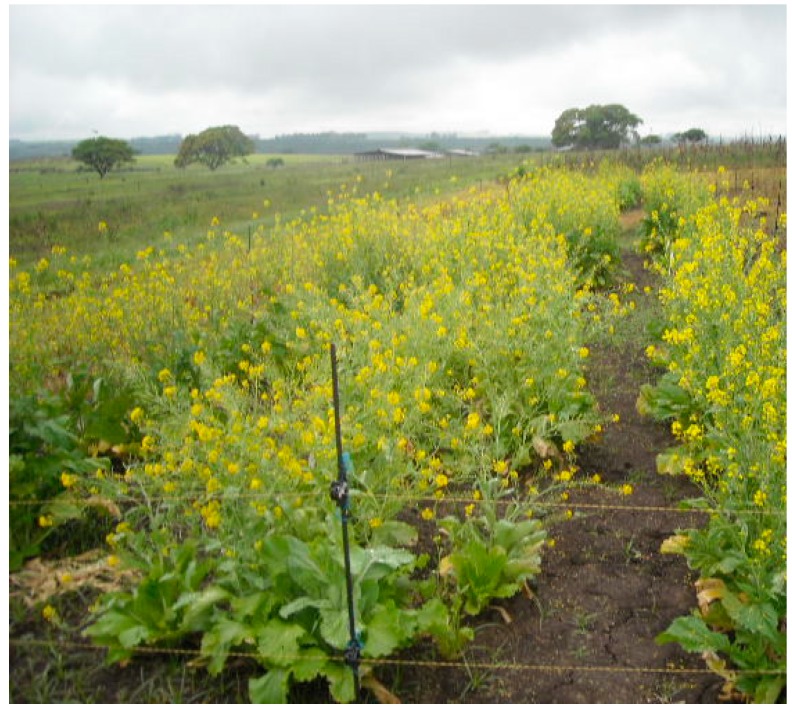
Wild mustard landraces. Source [10].

Wild mustard [*Brassica juncea* (L.) Czern & Coss and *Brassica nigra* (L.) W.D.J. Koch] (Figure 8) is an indigenous leafy vegetable of South Africa and belongs to the family of *Brassicaceae* or *Crucefereae* [101]. It is cultivated under diverse environmental conditions and is of great importance to the nutrition and livelihoods of SSA’s rural population. Wild mustard, like many other African leafy vegetables, provides essential vitamins, trace elements (iron and calcium) and other nutrients that are important for good health [102]. The seeds also have high oil and protein content [103], although this is dependent on environmental conditions [104].

Wild mustard has been reported to establish quickly, thus achieving optimum ground cover. According to Woods *et al.* [105], this growth characteristic is a good stress avoiding mechanism especially in water-scarce environments. Current information on the crops husbandry is locked up in IK systems and similar to other African leafy vegetables; there has been very limited scientific research on the crop.

#### 5.4.3 Wild Watermelon

**Figure 9:**
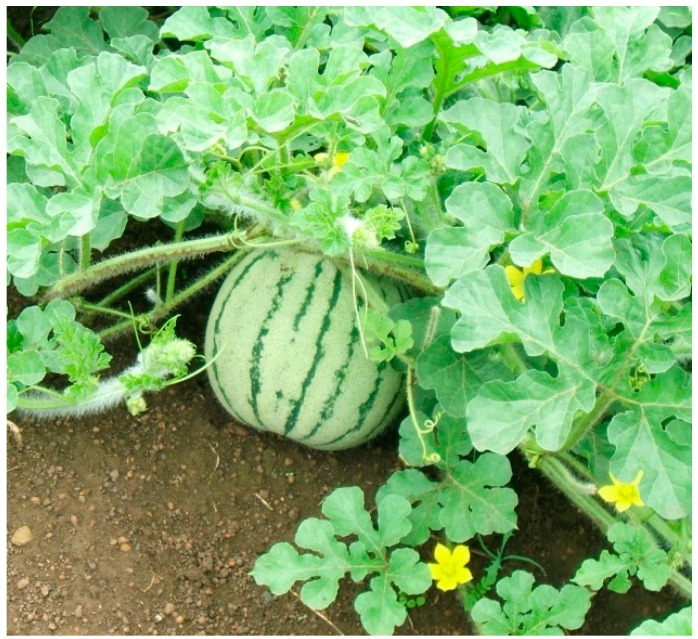
Wild watermelon. Source [10].

Wild watermelon (*Citrullus lanatus* L.) is a native crop of southern Africa (Figure 9). David Livingstone, an early explorer of Africa, described it as abundant in the Kalahari Desert, where it is believed to have originated. There, the ancestral melon grows wild and is known as the Tsamma melon (*Citrullus lanatus* var *citroides*) [106]. It is a vine-like plant or a climber and trailer herb, with edible fruits and leaves. The former name *Citrullus vulgaris* (vulgaris meaning “common”) [107] is now a synonym of the accepted scientific name for watermelon, *Citrullus lanatus*. It is regarded as the most morphologically diverse species in the genus *Cucumis* [108]. Varieties differ widely in fruit size, morphology and taste, as well as vegetative traits and climatic adaptation. Wild and early watermelons were extremely bitter, but this was eliminated quickly under cultivation with the selection of seed and cross-pollination.

Wild watermelon has a long history of cultivation and is grown throughout the world as a staple food (edible seeds and flesh), and for animal feed [109,110]. The rind is utilised for products such as pickles and preserves as well as for extraction of pectin [111,112], whereas seeds are a potential source of protein [110,113] and lipids [114]. The fruits are a popular and important source of water in the diet of the indigenous people in the Kalahari Desert during dry months of the year when no surface water is available.

The plant itself has been observed to be drought tolerant [10,115]. According to Miyake and Yokota [116] wild water melons keep their photosynthetic apparatus intact during prolonged drought. This would suggest that there are mechanisms present which make the plant tolerant to water deficits and excessive light energy falling on the leaves [117]. However, wild water-melon is still considered as a neglected and underutilised crop species; within the context of SSA, there is a dearth of information on agronomy and possible drought tolerance of diverse local landraces.

### 5.5. Indigenous/Wild Fruits

Forests and homesteads are important sources of non-timber products. These products include indigenous/wild fruits which are consumed by communities and also sold on road sides and urban markets to generate income. These fruits are essential for food security, nutrition and health, social and economic welfare of rural communities.

The miombo ecosystem of southern Africa is home to 200 species of fruits and 167 species are edible [118]. Fruits and products made from indigenous fruits constitute a cheap and yet rich nutritious source of food for which the poor depend on. Fruits and products from indigenous fruits are important during the hunger period of the year [118]. Indigenous fruits help women in rural communities to secure food for their families. They generate much needed income which will be used for various household uses including purchase of food. Miombo indigenous fruits such as *Uapaca kirkiana*, *Sclerocarya birrea*, *Strychnos cocculoides*, *Adansonia digitata* and *Parinari curetallifolia* are rich in sugars, essential vitamins, minerals, protein, carbohydrates and oils which are essential for human nutrition [119]. Domestication and commercialization of indigenous fruits to improve rural households’ nutritional status and income partly depend on IK systems of rural farmers. However, IK on these fruits varies according to tribe, between man and woman and between different ages. Women had more IK than man on leafy vegetables while men had more knowledge on indigenous fruits and edible roots. In urban areas knowledge on indigenous fruits and vegetables is usually limited especially amongst youth and young age groups.

While commercialization of indigenous fruits and vegetables seems to be underway, there is need for community involvement. There is also need to raise awareness amongst communities on intellectual property rights (right to access to their knowledge and landraces) and benefit sharing. The application of local community indigenous knowledge on indigenous fruits and vegetables such as their nutritional value will enhance their use and value of these underutilized crop species.

## 6. Sustainability of NUCS

The demands and expectations of modern supply chains lead farmers to concentrate on fewer and fewer crops, mostly handed in a top-down approach without consideration of IK and local communities [35], which has resulted in a steady loss of agro-biodiversity. This loss, if not corrected, will lead to irretrievable loss of strategic underutilized crop resources necessary for the wellbeing of millions of people, particularly those living in marginal areas. The rate ok loss of NUCS through extinction and genetic erosion is accelerating in many parts of the world as the result of drought, pest and diseases, over exploitation, over grazing, land clearance, deforestation and lack of incentives for farmers to maintain this agro biodiversity [27,120,121]. Together with loss of species, there is an accompanying and equally alarming wide spread erosion of local traditions and knowledge. Thus, for NUCS to play a significant role as future crops, there is a need to tap into IK, so as to incorporate local traditions and make production of NUCS relevant to local peoples [35]. Additionally, there is need for concerted efforts to promote on-farm genetic resource conservation of the NUCS given that the farmers have IK passed on from generations and there has been co-evolution of social and ecological systems at local levels [122]. This means that the sustainability of NUCS lies in the integration of IK and involvement of the local communities through local genetic conservation of NUCS.

However, while conservation of genetic resources in important for the sustainability of NUCS, breeding efforts are needed so at to improve the competitiveness of the different crop species and to make them adaptable to different climates [123,124,125]. It is not surprising that NUCS have been neglected in breeding programs, yet landraces have been widely utilized for genes that provide genetic resilience. Instead, breeding programs should focus on improving NUCS and make them more adaptable to the changing climate [27,126]. In addition, there is a need to develop value chains of different NUCS from the input side and the marketing of the produce [127]. Value chains for NUCS need to be developed so as to make them commercial products that can be traded not only on the local market, but also internationally [123,128]. This means that there is a need to promote the utilization of NUCS [129], coupled with value addition of the harvested crops. Consequently, sustainability of NUCS requires concerted efforts to improve utilization of the produce coupled with conservation of the genetic resource base, its genetic improvement and value chain development.

## 7. What Role(s) do NUCS Have to Play in the Future?

The combination of water scarcity, climate change and variability and increasing population that SSA is facing has painted a gloomy picture of future food security for a region that already has scarce water resources. The impending threat has led to previously NUCS being touted as possible future crops [19,22]. Decades of ‘neglect’ by researchers and farmers in favour of major crops have meant that NUCS have had to survive over the years, often under harsh conditions, without much assistance from man. As such, NUCS may have evolved to become adapted to adverse environmental conditions such as drought stress [78,83,130]. It is within this context that NUCS have a role to play as possible future crops. If indeed NUCS have evolved to become drought tolerant, they may have a role to play in guaranteeing future food security either directly as alternative crops in areas that are predicted to become drought-prone or indirectly as germplasm resources for crop improvement.

In addition to their adaptation to diverse ecological niches, most NUCS are said to be highly nutritious and in some cases to have medicinal properties. For example, African leafy vegetables have significant nutritional and health benefits compared to their exotic counterparts [131]. There is, however, limited quantitative information proving some of these claims. Some of the knowledge on the nutrition of NUCS remains hidden in IK systems and this may explain why certain communities have continued to preserve and utilise certain NUCS. Their unique adaptation and diverse uses speak to the role that they have historically played in rural communities. The fact that there is limited empirical information attesting to this serves to highlight the fact that NUCS remain under-researched. This speaks to the need for robust and comparable empirical data on aspects such as nutritional value of NUCS.

## 8. Conclusions

It is possible that the key to future food and nutrition security may very well lie in the untapped potential of NUCS. Therefore, it is imperative that we study locally available neglected underutilised crops and evaluate them for drought tolerance using agronomic techniques as well as modern techniques such as crop modelling, which allow for rapid evaluation of production scenarios. Since a crop’s ability to tolerate drought is dependent on a complex or dynamic variety and combination of responses and mechanisms, the commentary sought to evaluate the dynamics of drought tolerance in selected NUCS within the context of SSA. An understanding of morphological mechanisms involved in the responses of these NUCS is fundamental to their identification as drought tolerant crops. Such an understanding of morpho-anatomical responses would contribute significantly towards breeding for drought tolerance and making available developed varieties of these NUCS. The use of crop modelling as a technique may also aid in the interpretation of agronomic field data. Well–calibrated and validated models could also assist as selection tools for drought tolerance in these NUCS thus reducing on time and resources needed to fill the knowledge gap on these NUCS.
